# Automatic Speech Recognition Method Based on Deep Learning Approaches for Uzbek Language

**DOI:** 10.3390/s22103683

**Published:** 2022-05-12

**Authors:** Abdinabi Mukhamadiyev, Ilyos Khujayarov, Oybek Djuraev, Jinsoo Cho

**Affiliations:** 1 Department of Computer Engineering, Gachon University, Sujeong-gu, Seongnam-si 13120, Korea; mukhamadiyev@gachon.ac.kr; 2 Department of Information Technologies, Samarkand Branch of Tashkent University of Information Technologies Named after Muhammad al-Khwarizmi, Tashkent 140100, Uzbekistan; i.khujayorov@samtuit.uz; 3 Department of Hardware and Software of Control Systems in Telecommunication, Tashkent University of Information Technologies Named after Muhammad al-Khwarizmi, Tashkent 100084, Uzbekistan; od@tuit.uz

**Keywords:** convolutional neural network, end-to-end speech recognition, transformers, CTC-attention, Uzbek language, deep learning, hidden Markov model

## Abstract

Communication has been an important aspect of human life, civilization, and globalization for thousands of years. Biometric analysis, education, security, healthcare, and smart cities are only a few examples of speech recognition applications. Most studies have mainly concentrated on English, Spanish, Japanese, or Chinese, disregarding other low-resource languages, such as Uzbek, leaving their analysis open. In this paper, we propose an End-To-End Deep Neural Network-Hidden Markov Model speech recognition model and a hybrid Connectionist Temporal Classification (CTC)-attention network for the Uzbek language and its dialects. The proposed approach reduces training time and improves speech recognition accuracy by effectively using CTC objective function in attention model training. We evaluated the linguistic and lay-native speaker performances on the Uzbek language dataset, which was collected as a part of this study. Experimental results show that the proposed model achieved a word error rate of 14.3% using 207 h of recordings as an Uzbek language training dataset.

## 1. Introduction

Speech is the most basic and natural method of human communication and is capable of conveying important information both quickly and precisely. People are now committing the time and effort to learn how to communicate with diverse smart devices using vocal commands. The originality of speech has been discovered in 7097 living languages around the world [1]. A living language is “one that has at least one speaker who speaks it as their first language.” The large number of spoken languages does not imply that they are evenly distributed among the world’s population, and more than half of the population globally uses only 23 languages, including Chinese, English, Spanish, and Hindi. These are the most broadly spoken languages and are well covered with many data resources, making it possible to develop Artificial Intelligence (AI) based systems for Text-To-Speech (TTS), Automatic Speech Recognition (ASR), natural language processing, and computational linguistics. However, unpopular languages lack resources for specialized technology development and research. As a result, the significance of developing similar strategies for low-resource languages is a challenging and notable task.

ASR is an active and essential research area owing to its wide range of applications, such as security [2], education [3], smart healthcare [4,5], and smart cities [6], as well as the development of interfaces and computing instruments that can enable voice processing. It is a combination of various approaches that assist in the conversion of acoustic data into text, using text matching applied to the detected speech signal occurring in the result. The goal of ASR is to convert speech signals into text data by applying a stable basis for more in-depth semantic learning. Computer technology, digital signal processing, acoustics, AI, linguistics, statistics, and other fields are all included in ASR [7]. 

Tremendous advancements have recently been made in the field of ASR using different deep learning approaches. Although technological giants such as Google, Apple, Facebook, Microsoft, Amazon, and International Business Machines (IBM) have built advanced speech recognition engines for English, European, and Asian languages, research on ASR system development in most Central Asian languages such as Uzbek is still in its initial stages. This is due to the lack of a standard Uzbek language speech corpus, as well as dialectal differences [8]. First, Central Asian languages currently lack the minimum speech corpus required for the development of an ASR system. Second, a large-scale speech corpus can increase the computing complexity and dramatically reduce the performance in the testing step if a suitable categorization method is not first determined for the ASR system. Another key problem in developing an ASR system is the collection of a sufficient speech corpus and data for system training and testing. Out of the approximately 7000 languages used worldwide, such training data are currently available only for popular languages [9].

In addition, we consider that ASR will be the prospective method of human-machine communication Owing to the rapid development of their associated professions, the implementation of an ASR system has been substantially enhanced and extensively employed under different areas, such as social and medical services and the military, reducing human capital and enhancing job productivity [10]. All people have accents, and our voices vary significantly; thus, one of the main challenging tasks in creating an ASR system is overcoming such differences in speech patterns. Multilingual individuals have more differences in speaking accents than individuals who speak only one language. A similar challenge increases when additional elements, such as social habits, gender, dialects, and speaking speed, are added to an approach obtaining sufficient resources for teaching the ASR model.

Several voice assistants can currently recognize human speech patterns through an interactive real-time smart dialogue and apply automatic techniques based on the recognized content, such as Google’s Assistant and Apple’s Siri, which can converse in over 40 and 35 languages, respectively [11]. The majority of popular ASR systems use Gaussian Mixture Models (GMMs), Hidden Markov Models (HMMs), and Deep Neural Networks (DNNs) [12,13,14,15]. DNNs play an essential part in the building of ASR systems [16,17], mostly because of the evolution of unique neural network models, as well as training and classification techniques [18,19]. They have also been applied to problems such as feature extraction [20,21], audio signal classification [22,23], text recognition and TTS [24,25], disordered speech processing [26], and speech recognition based on small and large vocabulary [27]. Nevertheless, the speech datasets utilized to train DNN models have a significant impact on the performance of ASR systems.

In recent years, there has been a noticeable change in the ASR systems from DNN-based hybrid modeling [28] to End-To-End (E2E) modeling [29,30,31,32]. Hybrid models require disjoint optimization of separate part models such as pronunciation, acoustic, and language. At the same time, E2E ASR systems directly translate an input speech sequence into an output token sequence utilizing a single network, and the pronunciation, acoustic, and language modeling parts are trained together in a single system. 

Until now, the Uzbek language’s state-of-the-art ASR has been provided by modular Deep Neural Network Hidden Markov Model (DNN-HMM) systems. Recently, the most acceptable ASR results on Uzbek speech corpus data have been published [33], with WER of 17.4% on the Uzbek speech corpus test set. When creating speech recognition models for the Uzbek language, two significant obstacles exist:

- Uzbek is an agglutinative language. New words can be derived by adding many suffixes to the root of the word, the vocabulary size increases considerably;
- Existence of different Uzbek dialects with limited labeled data. Each dialect is a native Uzbek language that is spoken, but not written, as it does not have standardized orthographic rules.

To address these problems, we propose an ASR system based on the DNN-HMM model for the Uzbek language and its dialects. Hybrid Connectionist Temporal Classification (CTC) was applied as an E2E solution. The proposed approach reduces training time and improves speech recognition accuracy by effectively using CTC objective function in attention model training.

The main contributions of study are as follows:

- In a DNN acoustic model, a combined E2E and DNN-HMM hybrid ASR system was proposed using a shared network. To our knowledge, this is the first attempt to use deep-learning techniques to develop an E2E Uzbek language and dialects speech recognition system;
- Various state-of-the-art position-embedding methods were investigated using a convolution-augmented transformer for Uzbek speech recognition;
- Several state-of-the-art analytical techniques were presented for the hypothesis from the proposed ASR system to learn the Uzbek language, which leads to better performance and advancement of the overall system;
- We conducted experiments and achieved robust results on Uzbek language speech recognition using an E2E architecture and the DNN-HMM.

Additionally, this study contributes to developing a vocal activity detection (VAD) pipeline for the E2E transformer to handle the issue of very lengthy speech segments, which is a common occurrence in an experimental ASR system. The constructed pipeline combines the accuracy of the InaSpeechSegmenter with the maximum length of the energy-based VAD segmentation feature [34]. Finally, our main objective to create a new benchmark, complete with state-of-the-art recipes and pre-trained models, and make them accessible to the speech community.

The rest of this paper is arranged as follows. We review published popular ASR approaches in Section 2. Section 3 provides an understanding of the collected Uzbek speech corpus and the proposed ASR system for Uzbek. Section 4 and Section 5 describe the implementation and evaluation of the proposed system using other cutting-edge approaches. Section 6 discusses the limitations of the system and possible future directions. Section 7 gives the main findings and concludes the paper.

## 2. Related Work

For decades, HMM- and GMM-based models have dominated the speech recognition field. The number of states created throughout the training process is fixed and predefined in the HMM, which is a stochastic model. The number of hidden states in an incoming speech signal may differ between these states. The HMM assumes that the input speech signal may be described as a parametric random procedure with well-defined and accurate parameters. By contrast, DNN models have now become a crucial feature of TTS and ASR [35,36], and different speech analysis and recognition problems, resulting in a significant advance in speech technology in recent years. Compared with traditional artificial neural networks (ANNs), DNNs include more than one hidden layer between the input and output layers. The neuron unit is the most fundamental element of a DNN. To create a fully connected network, the neurons in each layer are completely linked to the neurons in the neighboring layers. Acoustic models based on DNNs have nearly superseded HMMs and GMMs in ASR systems. However, the structural locality from the feature distance is not captured because of the fully connected characteristic of the DNN, which is a major shortcoming. Second, when using a Stochastic Gradient Descent (SGD) for training, the DNN encounters a vanishing gradient issue [37].

In addition, instead of hand-engineered features, researchers have begun to use raw speech. They have collected data-driven components associated with the task at hand using DNN models for these features [38]. Various speech-connected problems such as emotion detection [39], ASR [40], and speaker identification [41] have demonstrated encouraging results using such attributes. Researchers in the field of speech technology have recently concentrated on Convolutional Neural Networks (CNNs), effectively applied in E2E models that are trained using raw speech datasets. Such networks can train a filter bank from raw speech and use the raw waveform to detect better discriminative, contextual, and generic representations [38]. CNNs have been demonstrated to successfully simulate structural locality in the feature space as well as employ pooling within a biased frequency region to reduce translational variation and adjust for disturbances and small changes in the feature space [42]. By manipulating the prior knowledge of the speech signal, they may take advantage of the lengthy connections between speech frames. However, Soltau et al. [43] reasoned that CNNs in ASR systems do not absorb stress for semi-clean data; as a result, the performance of the system suffers.

RNNs, however, improve the identification accuracy by collecting extended contexts, which is especially useful for noise-resistant applications. However, the capacity of RNNs to learn the time dependencies is limited by the vanishing and exploding gradient issues. To address these issues, long short-term memory (LSTM) was developed, which uses a specific unit called a memory block to manage the flow of data [42]. LSTM-RNNs are liable to static input, therefore, target pauses occur in terms of the features. LSTM is a complicated network unit with a memory function that allows it to store data for lengthy periods. There are three types of gates in the LSTM framework that can selectively recall historical data: input, forget, and output gates. The input entry controls whether or not input is permitted in the cell unit, the forget entry controls whether or not the memory of the prior point is memorized, and the output entry controls whether or not the memory is allowed to flow to the following point. For acoustic modeling, a low delay between the inputs and outputs is preferable. Consequently, a specific architecture known as bidirectional LSTM (BLSTM) was created, which processes the input series in both paths to produce determinations. 

There has been an increase in interest in developing E2E-Trained ASR models that can directly translate the input acoustic speech signal to a word sequence or grapheme. Researchers are conducting extensive research into E2E ASR systems at Google and Microsoft, like other giants of the information technology industry. In 2017, Prabhavalkar et al. [29] explored various sequence-to-sequence techniques, including a CTC-trained system that outputs grapheme sequences directly, the RNN transducer (RNN-T), attention-based models, and a new model that augments the RNN-T with attention. Their research demonstrated that when trained on such a massive amount of training data, approximately 12,500 h, sequence-to-sequence techniques are competitive on dictation test sets with a robust state-of-the-art baseline system. In the same year, Rao et al. [30] evaluated the performance of a sequence-to-sequence RNN-T. This model consists of an encoder that is initialized from a CTC acoustic model and a decoder that is partially initialized from an RNN language model trained on text data alone. Text and pronunciation data can be used to improve E2E ASR performance. They discovered that pre-training the RNN-T encoder with CTC improves WER by 5% relative to a 5-layer encoder and that employing a deeper 8-layer encoder rather than a 5-layer encoder improves WER by 10% compared to a 5-layer encoder. In 2019, Google researchers, He et al. [31], introduced the E2E ASR, which operates twice as fast as real-time on a Google Pixel phone and improves WER by more than 20% over a powerful embedded baseline system for both voice search and dictation tasks. In 2020, Li et al. [32], Microsoft Speech and Language Group researchers, created an RNN-T model encoder using CTC or Cross-Entropy (CE) training. The model was trained using 65,000 h of transcribed Microsoft data, and the CE initialization of the RNN-T encoder lowered WER by 11.6% relative to the zero-lookahead model, while the model with future context increased by 12.8% relative to the zero-lookahead model.

Several types of studies have been conducted to recognize speech in various languages, but only a few have been applied to Central Asian languages. Mamyrbayev et al. [44] introduced an ASR system using a DNN model and Kaldi tools for Kazakh. Later, in 2021, the authors of [44] proposed an E2E ASR model using an RNN-T, that has a similar network as an encoder–decoder with an attention architecture [45], to recognize Kazakh language speech. This system consists of three parts, encoder, prediction, and joint networks, and was trained using 300 h of the Kazakh speech corpus. However, this 300 h of the Kazakh speech corpus is not publicly available and is small for training robust E2E ASR models. To solve these limitations, Khassanov et al. [46] created an open-source speech corpus for the Kazakh language that contains approximately 332 h of transcribed audio and over 153,000 utterances uttered by people of all ages and genders, and several geographical locations. They began by extracting Kazakh textual data from a variety of sources, including legislation, electronic publications, and websites, such as Wikipedia and blogs. They then narrated the extracted lines using a web-based speech-recording technology that could be utilized on both personal computers and smartphones. Earlier efforts focused on the creation of Uzbek speech recognition in the context of the Uzbek language. For example, an initial Uzbek speech corpus consisting of 3500 utterances [8] was developed. In 2021, Musaev et al. [33] proposed an HMM and DNN-based ASR system for the Uzbek language. They used 108,000 utterances to train the proposed model, yielding around 105 h of transcribed speech data. Other Uzbek speech corpus datasets are either excessively expensive or inaccessible to the public. As a result, the creation of a sufficiently large open-source Uzbek speech corpus and an ASR system is critical.

## 3. Materials and Methods

In this section, the details of the proposed model are described. First, we provide the architecture of the Uzbek language ASR system in Section 3.1. We then describe the speech corpus preparation for model training in Section 3.2. One of the most important factors in creating an adequate and successful deep learning model is correctly preparing the speech corpus. For the deep learning model to be successful, the number of audio utterances and their transcriptions in the training corpus must be large and gained under diverse requirements. Section 3.3 describes the acoustic and language modeling for decoding. The E2E transformer model applied to the proposed ASR system is described in Section 3.4. We used CTC attention to reduce loss by ordering the correct transcriptions with greater probability, as represented in Section 3.5. In Section 3.6, we give a detailed explanation for the encoder and decoder generating text based on these distributed probabilities.

### 3.1. ASR System Architecture for Uzbek Language

The modeling of a speech recognition system involves the collection of audio utterances, the transcription of audio files, the mapping of letters to sounds used to form phoneme dictionaries, phonetic transcription, acoustic modeling, language modeling, and the extraction of speech features, and a learning algorithm with loss minimization. Instead of relying on phonetic transcription or synced audio samples with letter-to-phoneme mapping, the E2E speech recognition methodology is the requirement for these steps. As an alternative, a raw audio file is provided to the ASR system, which extracts audio properties, maps audio segments into characters, and delivers the identified characters together with their probability of being recognized. MFCC characteristics are extracted from audio recordings and fed into a six-layer deep neural network, which calculates the probability for all alphabets using the deep speech method. A language model is then used to translate these probabilities into intelligible words and phrases, ultimately presented to the audience.

**Figure 1:**
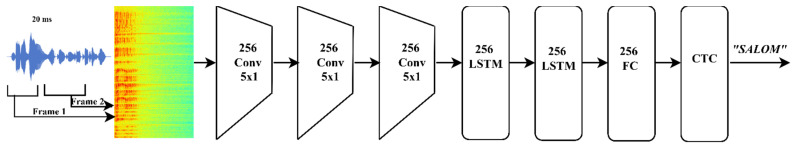
The proposed network architecture.

The proposed network architecture is described in Figure 1. The 40 × 1 vector values of the Mel-spectrogram from the speech fragment every 20 ms to the network architecture are passed to the convolutional layer as input. At the beginning of the network are three consecutive layers of convolution. Bi-directional LSTM layers follow this. After this recurrent layer, fully bonded layers were placed, and the softmax activation function was applied. The layer parameters in the network architecture are listed in Table 1. 

**Figure 2:**
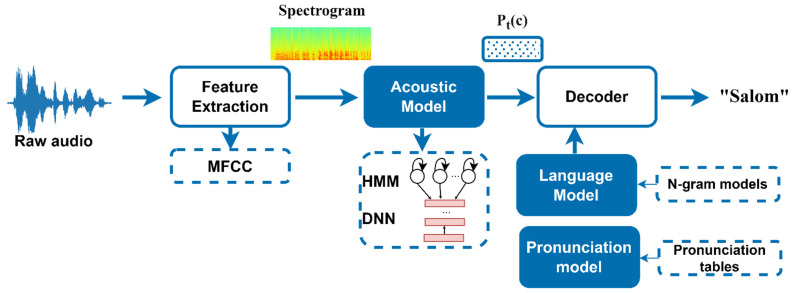
Overview of the proposed Uzbek language ASR system.

The creation and optimization of automatic speech recognition are described in Figure 2. ASR is a difficult problem in natural language because it entails a number of subtasks such as speech segmentation, acoustic modeling, and language modeling to generate a prediction (of label sequences) from noisy, unsegmented input data. However, the emergence of CTC eliminates the necessity for pre-segmented data and allows E2E training of the network for sequence-labeling tasks such as ASR. 

Consequently, the following blocks comprise a CTC-based ASR pipeline:

1. **Feature extraction**: Normalization, windowing, and (log) spectrograms are used to preprocess audio signals (or a Mel scale spectrogram or MFCC);
2. **Acoustic Model**: A CTC-based network is applied that predicts the probability distributions *P_t_(c)* over vocabulary characters *c* for each time step *t*;
3. **Decoding**:
  - The greedy method (argmax) is the simplest decoding method. At each time step, the letter with the highest probability (temporal softmax output layer) is selected regardless of any semantic comprehension of what was transmitted. The repeated characters are deleted or compressed, and the empty tokens are discarded;
  - A language model can offer context for the acoustic model, which can help to remedy any inaccuracies that may have occurred. Using a beam search decoder, try to decode what was said by integrating what the acoustic model believes it heard with the likelihood of the following word occurring in the context.

### 3.2. Uzbek Language Speech Corpus

The first important task in speech recognition is data collection and processing. The training corpus comprises audio utterances and their transcriptions. A corpus of speech includes read, spoken, or spontaneous communication. The following procedures must be conducted to construct a corpus of reading speech: (1) Create vast text data (sentences) that cover the majority of the target language vocabulary, and (2) record these lines in a variety of contexts (loud, clean, and outside/inside) by various speakers (native, fluent, and of varying ages and genders). A conversation corpus can be built by recording and transcribing a speaker’s usual discussions on any subject. Typically, these raw recordings are referred to as uncleaned records owing to the fact that they require a post-processing to generate a clean record. The post-processing stages include producing transcriptions of audio utterances, cleaning up audio samples (removing excessively loud or repeated phrases, for example), and synchronizing audio and text transcriptions. The entire procedure is time-consuming and costly (e.g., setting up the recording, incentivizing the speakers, and hosting the data). Researchers with limited money and resources must rely on publicly available corpora or novel crowdsourcing techniques [47].

Nevertheless, an excellent open-source approach applied by Mozilla [48] for developing free voice corpora, the Common Voice Corpus 8.0, has captured speech data for 14,000 h in 87 languages, making it a valuable resource. The Uzbek language is represented by more than 227 h of recorded speech in the Common Voice Corpus 8.0, of which only 80 h have been validated. A generic large-vocabulary Uzbek speech corpus has not been generated and made available to the general public for research purposes yet. The Common Voice and SpeechOcean speech corpora were employed to train the Uzbek automated speech recognition system using natural language processing. A summary of the two corpora is provided in Table 2, which includes the total amount of words, the total number of unique words, the total duration, the total number of utterances, the average duration of each utterance, and the average number of words per utterance.

### 3.3. Modeling of Acoustic and Language

This study used the architecture proposed by Hussein et al. [49], which integrates a time-delay neural network (TDNN) with LSTM layers and delivers significantly better results than BLSTM acoustic modeling. The TDNN-LSTM model is comprised of six hidden layers, each of which has 1024 hidden units in total. [50] The neural networks were trained using the lattice-free maximum mutual information (LF-MMI) technique [50]. MFCC features devoid of energy and their first and second derivatives are accepted as input by the modular system in the form of typical 13-dimensional cepstral mean-variance normalized (CMVN) MFCC features devoid of energy. A linear discriminative analysis was performed to project the concatenated frames into 40 dimensions, followed by a maximum likelihood linear transform to ensure each frame had four adjacent frames. In the feature space, speaker adaption was also utilized in conjunction with maximum likelihood linear regression (fMLLR). Five thousand states are represented by 100,000 Gaussians in the GMM-HMM model. Concerning the development set, the language weight and quiet penalty parameters that provided the most exceptional performance were 0.8 and 0.0, respectively. The acoustic models were constructed using the Kaldi ASR toolkit [51]. 

We trained two n-gram LMs.: a big 4-gram LM (bLM4) trained on spoken transcripts and the 9M-word background text, and a smaller 4-gram LM, which was pruned using pocolm. A small LM was used to produce lattices using first-pass acoustic decoding. Subsequently, bLM4 was applied to rescore these lattices. To maintain consistency with the E2E system, the time-restricted self-attention layer proposed was employed in [52] for acoustic modeling. In addition, we investigated sub-word modeling with 128,000 subwords that had been adjusted for the DNN-HMM. Our findings indicate that word tokenization resulted in a 1.1% relative decrease in word error rate (WER) on the Uzbek development set and a 0.7% relative increase in WER on the Uzbek_test set. As a result, we opted to abandon subword modeling and instead use the cutting-edge TDNN–LSTM architecture with word tokenization. This modular system is referred to as DNN-HMM throughout the study.

### 3.4. The End-to-End Transformer

**Figure 3:**
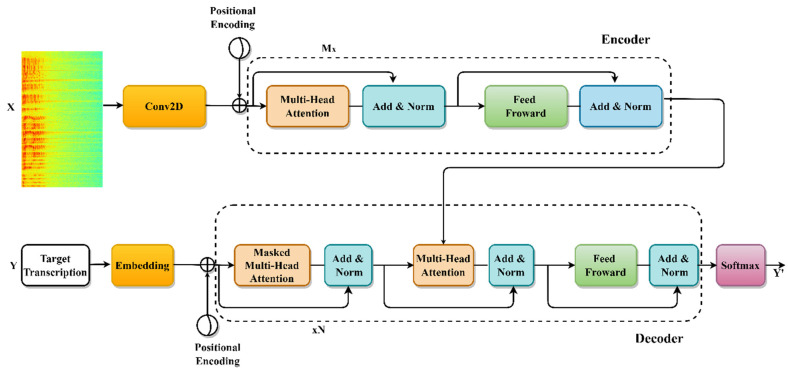
ASR architecture based on E2E transformers. During training, the encoder is fed a sequence of feature frames. In addition, the encoder output is passed to the decoder for the masked target transcription. The decoder provides a prediction of the masked transcription.

A transformer is a modern sequence-to-sequence model that fully eliminates recursion in standard recurrent networks, in favor of a self-observation mechanism and sinusoidal position information. As shown in Figure 3, we adopted a transformer-based design to represent the Uzbek ASR. 

A transformer has M repeating encoder blocks and N repeating decode blocks. Maps of the encoder model and vector of input to its hidden representation. There are Log-Mel spectrograms with 80 dimensions and Feature frames of 83 dimensions sent into the X encoder [53]. The decoder makes one prediction at a time. The encoder model’s latent representation and previous decoder predictions are given at each step.

### 3.5. Hybrit CTC-Attention Architecture

CTC was employed to reduce the loss by ranking the accurate transcriptions with a greater probability. A CTC output was created for each and every value of x in the input. There is a multi-frame output in the Uzbek language. To solve this problem, the CTC uses empty symbols to define the output’s border. For the encoder output vector h, which represents the encoder network output [54], CTC is utilized. The CTC is calculated using Equation (1):

(1) $$y^{*}=argmax_{y}P(y|h)$$

where y* is closest to *y* for the encoder output *h.P(y|h)*, which is calculated using (2):

(2) $$P\left(y|h\right)\approx \prod_{t=1}^{T}P\left(y_{t}|x\right)=\prod_{t=1}^{T}q_{t}\left(y_{t}\right)$$

In the network’s output layer, CTC is a loss function used to find an output sequence (2). The softmax activation value of $y_{t}$ in the encoder layer *q* at time *t* is given by the expression $q_{t}\left(y_{t}\right)$. This formula includes a loss function for CTC (3).

(3) $$L_{CTC}=-lnP\left(y|x\right)=-ln\sum_{t=0}^{T}\frac{\alpha_{t}\beta_{t}}{q_{t}}$$

**Figure 4:**
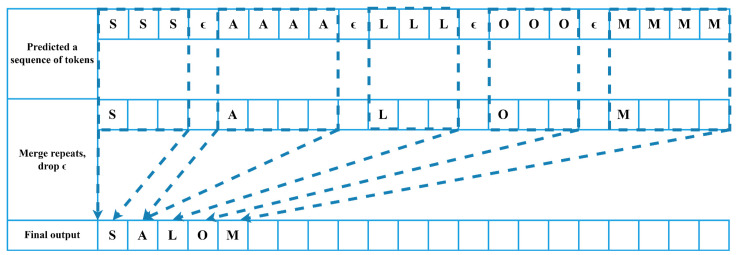
CTC networks were employed in this research for speech recognition [40]. CTC more reliably translates the expected sequence of tokens to valid words. The delay and repeated tokens in the anticipated sequence “sss aaaa lll ooo mmmm” are removed, leaving the final output as “salom” with the greatest probability.

The forward and backward approach is used to determine the CTC loss function. The forward value α is the total of all CTC training paths from *0* to *t*. The backward value *β* is the total of the ways between time *T* and time *t* in which the forward value exists. The CTC algorithm is shown in Figure 4. The deep neural network (feed-forward neural network (FFNN), RNN) generates a syllable or phoneme sequence. Alignment is the second phase. A blank sign is discarded, and a repeating character is combined to ensure alignment. Finally, the output is allocated to the combined character or phoneme.

**Figure 5:**
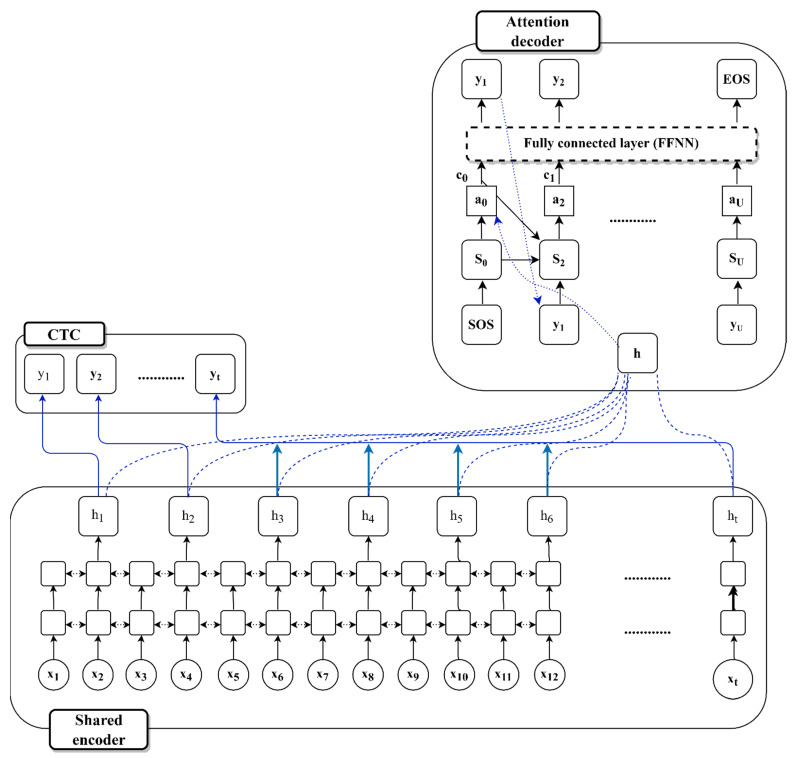
A network combining connectionist temporal classification (CTC) and attention-based voice recognition. The shared encoder transforms the input x into the encoder vector h as the output. Both the CTC and the attention models are trained on the common encoder. The probability distribution for the output y is generated using CTC and attention.

The structure of the hybrid CTC-attention network for the Uzbek E2E model applied to speech recognition is shown in Figure 5.

The input feature sequence is given by x, whilst the transcription of the input feature sequence is shown by y. The frame time of each x element is 10 ms. The length of the output sequence, on the other hand, is usually less than x. In the encoder–decoder model, the CTC and attention share the encoder. The attention weight vectors *a* and context vector c are created using the encoder output vector h. A bidirectional LSTM network structure is used in the encoder [55].

### 3.6. Encoder and Decoder

**Figure 6:**
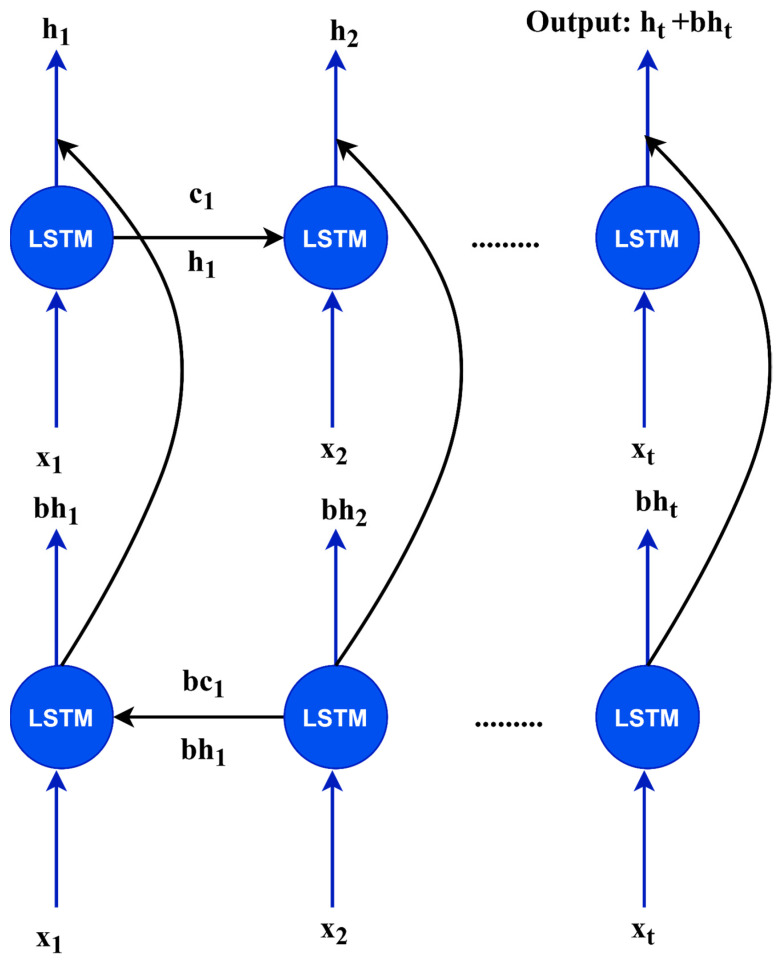
The architecture of bidirectional LSTM.

The number of inputs was significantly greater than the number of outputs in the voice recognition tasks. Multiple vector sequences are used to describe speech signals using feature extractors such as a perceptual linear prediction and MFCCs. Each vector sequence was approximately 5–10 ms in duration. For instance, a phoneme *[t]* has an average of 4 to 10 characteristics. To compress the input vector, the encoder converts speech feature vector *x* into encoder vector *h*. The encoder vector *h* is utilized as the input criteria of the decoder, which is referred to as an “attention decoders.” We employed a bidirectional LSTM–RNN as the encoder network in this study [56]. In deep-learning tasks, an LSTM–RNN network was utilized to tackle the sequential issues. A network refers to a large number of additional inputs to quickly create the encoder vectors, therefore, this recurrent network refers not only to the next node weight, but also to the previous one. The bidirectional LSTM is shown in Figure 6. In this case, *x*_1_, *x*_2_, and *x_t_* are inputs from the speech signal; *h*_1_, *h*_2_, and *h_t_* are from the forward LSTM layers; *bh*_1_, *bh*_2_, and *bh_t_* are from backward LSTM layers; and *c*_1_, *c*_2_, *c_t_*, *bc*_1_, *bc*_2_, and *bc_t_* are the values from the memory cells in each LSTM layer.

In speech recognition, neural networks are typically trained using individual fragments of speech signals. To do this, we must select the appropriate tabs for each frame, which makes it necessary to equalize the soundtrack and transcription. Decoding algorithms generate text based on these distributed probabilities.

## 4. Experiment

A dataset of Uzbek language spelling rules was used to evaluate the suggested E2E transformer methodology with current solutions. Conventional metrics of the WER and character error rate (CER) were used to evaluate the Uzbek language dataset.

Experimental studies have been conducted based on detailed learning algorithms for automatic recognition of the Uzbek language using integrated neural networks and the recognition of Uzbek language commands in the form of images on CNNs.

To conduct a complete evaluation of the ASR systems, we compared the conventional hybrid-based model with both the DNN-HMM described above and the E2E architectures. For the experiments, Uzbek language dataset was used for the experiments that included both the training and test sets.

Training of the speech samples prepared in the proposed network architecture was conducted on a Python Jupyter notebook. Our studies were conducted on high-performance computing (HPC) node equipped with four NVIDIA Tesla V100 GPUs with 16 GB of RAM and a 20-core Xeon(R) E5-2690 CPU.

### 4.1. Data for Model Development

In this study, the Uzbek language corpus was used for the model training. The dataset includes more than 2 years of recordings, from 2020 to 2021, with a total length of approximately 207 h. Conversations (63%), reports (18%), and interviews (19%), were included in the programs.

Additionally, the dataset included a large corpus of background text for use in developing a language model. Over 180,810 utterances, 904,230 words, and 128,860 unique words were included in the text corpus. Table 3 shows a detailed specification of the Uzbek language dataset.

This study compares the ASR of the proposed E2E transformer with the real-world Uzbek dialect dataset. The Uzbek dataset consists of 207 h of transcribed audio spoken by 1281 speakers. Native speakers carefully reviewed the corpus to ensure its quality. In addition, the study data were supplemented by data consisting of 52 h of audio files and the text transcripts of audiobooks by modern Uzbek writers.

### 4.2. Data Pre-Processing

We initially used a speed perturbation technique to our raw audio, which boosted the original signal by three times using speed factors of 0.9, 1, and 1, respectively [57]. E2E model feature frames were generated for each added sound and an 80-dimensional log-Mel spectrogram with pitch information was generated for each of the enhanced audio files. There were also three additional deformations added to the resultant Mel-spectrogram features: warping the features in the time direction, masking blocks of consecutive frequency channels, and masking blocks of utterances. For the creation of the language model, data from the transcription and 128,000 words of background text were both included [58]. All punctuation, diacritical marks, additional whitespace, newlines, and single-character words were removed from the data. In order to avoid the issue of very lengthy sequences, the text was split such that each section included a maximum of 200 words with a 50-word overlap [59]. Tokenizing the input text and creating the vocabulary were accomplished via the usage of the sub-word model [60].

### 4.3. Default Model Hyperparameters

A grid search was used to determine all hyperparameters. For parameter adjustment, a small portion of Uzbek data (207 h) was employed. Using the Adam optimizer, a model based on the E2E transformer was trained at a learning rate of 0.001. Twelve encoders and six decoders were used in the model. Both self-attention and encoder–decoder attention have four heads. The 2D CNN front-end used two 3 × 3 convolutional layers with 256 channels. Subsequently, the rectified linear unit (ReLU) activation function and a 2 × 2 max pooling operation were used. The hidden dimensions of the attention layer numbered 256. The hidden dimensions and output dimensions of the feedforward numbered 256 and 2048, respectively. The adaptive moment estimation (Adam) algorithm was used as an optimization method for the initial learning rate, the number of cycles was 100, and the batch size for each image was 10. SpecAugment was used for data augmentation. The CTC weight for the joint training with the attention model was set to 0.3. During the test phase, the CTC weight for the joint decoding was set to 0.6. A transformer-based language model was used to refine the results. Table 4 summarizes the best set of parameters that were found for the acoustic modeling (AM) and language model (LM) transformer architecture.

## 5. Experimental Results and Discussion

With the help of an expert linguist and a state-of-art modular system, the E2E transformer (E2E − T) was benchmarked. We also examined the contributions of CTC and attention objectives to the E2E − T overall performance. E2E − T (CTC + Attention) is referred to simply as E2E − T in the following. Table 5 summarizes the results of WER for the Uzbek_Test set and Hidden_Test set. The results of the WER were mapped from a digitized form into literal transcriptions using global map scoring (GLM). In terms of WER, the suggested E2E transformer with hybrid CTC + Attention surpasses the single DNN-HMM by 20%

Furthermore, the attention goal, which achieved a WER of 16.4% and 17.6% on the Uzbek_Test and Hidden_Test sets, respectively, made up the majority of the contribution to the total E2E − T performance. On the Uzbek_Test and Hidden_Test sets, adding CTC improves the WER by 2.1% and 3.2%, respectively.

### 5.1. Analyzing Human and Mechanical Errors

The following section will examine the mistakes and their correlations with expert linguists (linguists), native speakers (natives), the E2E transformer, and HMM-DNN ASR systems.

As one aspect common to all approaches, 56 instructors (28 men and 28 women) participated in the training of the speech corpus. Each narrator created a 38-min audio file based on the reading of stories, and novels by various authors of modern Uzbek literature. The total volume of the audio files was 22 h.

There were 124,368 words in the training speech corpus, of which, 42,415 were non-repetitive. We applied 80% of the entire speech corpus for training, 10% for the network setting during training, and 10% for testing.

**Figure 7:**
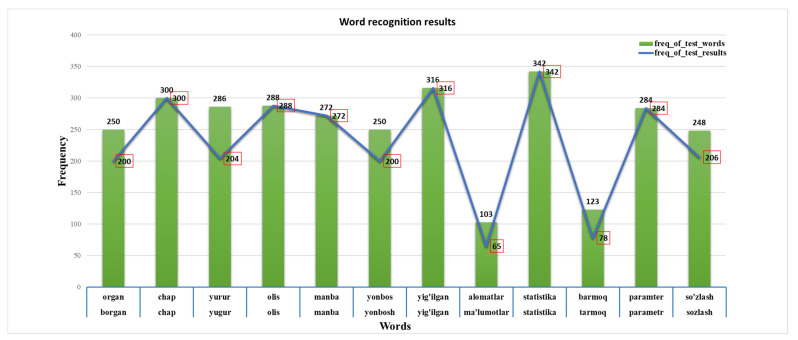
The experimental results of the proposed model on word recognition in the Uzbek language. Some words have more than 50 frequency errors.

Example results of the proposed automatic speech recognition model for Uzbek based on the integrated automatic speech recognition model are shown in Figure 7. In the experiments, we conducted a 22 h training session on the proposed neural network architecture. The set of words selected for testing had the highest frequency in the training speech corpus. Therefore, their automatic recognition was better than that for other rare words. Note that, the result of word recognition frequency was marked with red box.

**Figure 8:**
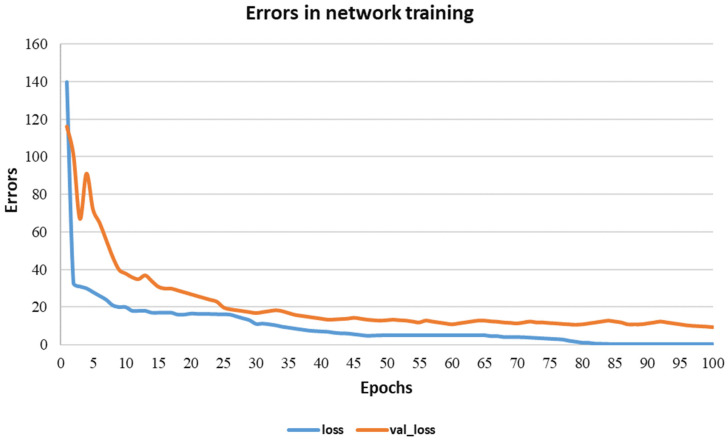
The training processes of the proposed model. Training loss and validation loss during network training.

We can see in Figure 8 that errors occurred in the word recognition of the system. In this case, errors were mainly caused by the casting of the appropriate sign. However, in most cases, it can be seen that the system correctly recognizes words when their frequency in the sample is greater than 100.

The test results were calculated based on the words recognized by the error. The evaluation metric for the number of incorrectly identified characters in the test was a CER of 22.7%. Ratings were obtained using a large dictionary. Better results can be achieved by the proposed network architecture if we implement the teaching of words in a limited dictionary.

We also considered the proposed approach when applied to a limited dictionary. Additional audio data were gathered to address the gaps that were apparent in the data. A total of 50 instructors (20 men and 30 women) participated in the selection process. Each announcer created an audio file based on the pronunciation of words. The total number of audio files used numbered 18,700. Eighty percent of the training sample was for training, 10% was for the network setting, and 10% was for testing. When evaluating the test results, the evaluation metric for the number of characters that were incorrectly identified during the test was a CER of 5.41%.

As shown in Figure 8, the errors in network training and testing depended on the size of the sample, the number of speakers, the speed of pronunciation in the creation of audio files, the acoustic field, and many other parameters, training using a limited dictionary, or the high-precision automatic recognition of arbitrary words. Depending on the size of the sample, the number of speakers, the speed of pronunciation in the creation of audio files, the acoustic field and many other parameters, the training in the limited dictionary or high-precision automatic recognition of arbitrary words.

### 5.2. Speech Recognition Results for End-to-End Transformer and DNN-HMM

This section analyzes the E2E transformer and DNN-HMM results and then shows the benefits and drawbacks of the two systems. In the samples, the accurate transcripts are indicated in green, while the equivalent mistakes are highlighted in blue.

1. **Dialectal Uzbek:** E2E produces a more accurate transcription for dialects and overlapping speech. Through the mechanism of self-observation, the E2E transformer can learn the context much more effectively and capture semantic information in both standard Uzbek and the various Uzbek dialects.
  - **Input Uzbek sentence:***Yangiliklardan doimiy xabardor bo’lib turish uchun kanalimizga a’zo bo’lib oling*;
  - **Correct output:***Yangiliklardan doimiy xabardor bo>lib qolish uchun kanalimizda a>zo bo>lib oling*;
  - **English Translation:** Subscribe to our channel to stay up-to-date with the latest news;
  - **Results of E2E**: *Yangiliklardan doimiy xabardor bo>lib qolish uchun kanalimizda azo bo>lib oling*;
  - **Results of DNN-HMM:***Yangiliklardan doim$> xabad’< bo>lsa qolish uchun kanalining zada bo>lsa qoling*;
2. **Noise and hesitation:** Noise and hesitation are more likely to be detected by the E2E transformer than by the DNN-HMM. Random words are generated when someone utters “uuuuh” or a bird chirps. The DNN-HMM has been shown to be more robust to noise and sounds of hesitation owing to the use of symbols in Viterbi matching during the training process. A similar symbol was used for the spaces in E2E with CTC loss. To improve the robustness, more data or the explicit labeling of noise and sounds of hesitation are required. The E2E optimization is more data-based than the DNN-HMM, which is based on expert knowledge.
  f. **Input Uzbek sentence**: *<bird_chirps> Hozir yuqoridagi savollarga javob berishga harakat qilamiz*;
  g. **Correct output:***<bird_chirps> Hozir yuqoridagi> savollarga javo$b berishga hara$kat qilamiz*;
  h. **English Translation:** We will now try to answer the above questions;
  i. **Results of E2E:***chu$chg >ug$ chu$>zir yuqoridagi> savollarga javo$b berishga hara$kat qilamiz*;
  j. **Results of DNN-HMM:***Hozir yuqoridagi> savollarga javo$b berishga hara$kat qilamiz*.

### 5.3. The Influence of Data Size on the Performance of E2E Transformer and ASR

**Figure 9:**
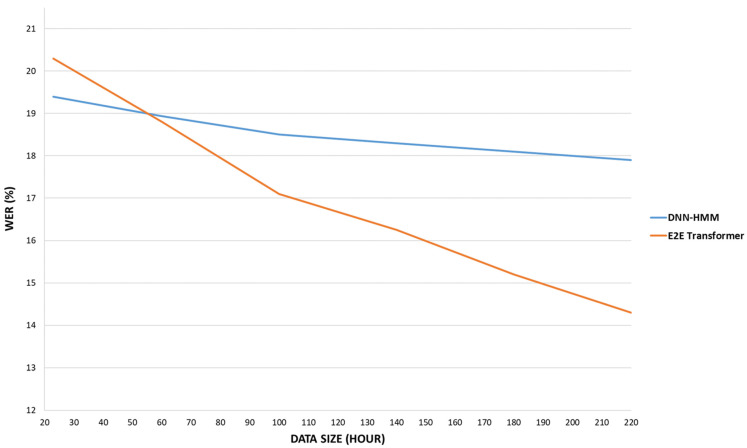
The WER measurements of DNN-HMM and E2E transformer performance were compared in various training data sizes.

In this sub-section, we compared the proposed modular systems such as E2E transformer and modular ASR system which is DNN-HMM and discussed the impact of data size on them. The default model hyperparameter for the two modular systems, namely the E2E transformer and the DNN-HMM were described in Section 4.3. The length of the training data was either 25, 110, or 207 h. In order to maintain consistency across training data sizes, the development and test data were kept constant. Figure 9 illustrates the performance of the E2E transformer and modular ASR system which is DNN-HMM for various data sizes. The modular ASR system performs much better when the data size is smaller than 60 h. However, as data size increase, the E2E transformer performs better than the modular ASR system, showing that future advancements in the E2E transformer model are possible. Furthermore, the findings demonstrate that the E2E transformer model surpasses the modular ASR system after 60 h of training data. Although 60 h of data may not seem like much for the Uzbek language, it represents a massive amount of transcribed material for most of the world’s popular languages and dialects.

As shown in Figure 9, DNN-HMM and E2E transformer models achieved 19.4% and 20.3% WER, respectively, when 20 h of training data were used. The DNN-HMM model achieved better results than the E2E transformer with up to 60 h of training data. The E2E transformer model achieved a 1% improvement in WER results with training data increasing every 20 h, resulting in a 14.3% WER with 207 h of training data. On the other hand, the increase in training data had almost no effect on the WER result of the DNN-HMM model, with an improvement of only 1.5% resulting in a WER of 17.9% with 207 h of training data.

### 5.4. LM Ablation Analysis of E2E Transformer

We explore the influence of LM on the acoustic E2E transformer performance in this section by utilizing transcription (TR) and background (BG) text. As demonstrated in Table 6, the acoustic E2E transformer’s WER (percent) and real-time factor (RT) are summed for both the RNN LM and transformer LM designs. The WER decreases as the beam size is lowered and the RT increases. An interesting point to note here is that the E2E model without LM achieves a better WER than E2E with an LM architecture.

The proposed E2E − T model is compared with other Uzbek language ASR models [61], as shown in Table 7. The comparison results showed that the proposed E2E − T model achieved robust results compared to Transformer [61] with WER 14.3 % and 19%, respectively.

We also compare the proposed E2E − T (CTC + Attention) model with other model in various scenarios with or without language model (LM), speed perturbation (SP), and spectral augmentation (SA). Table 8 shows the CER and WER results of E2E − T (CTC + Attention), E2E-Conformer, E2E Transformer, RNN-CTC, DNN-HMM, and E2E-LSTM models for the validation and test sets of the Uzbek language speech corpus. The proposed E2E − T (CTC + Attention) model achieved the most robust results than other models in all scenarios. We found that adding LMs to E2E ASR improved WER in the by 7.5%–12.75% on the test set after incorporating LMs. The E2E ASR models get additional whole WER improvements of 0.7%-6.2% when speed perturbation is applied to the test set. Spectral augmentation improves E2E ASR models on the test set by 1.8%-4.1%. However, it has no impact on the DNN-HMM model. The E2E − T (CTC + Attention) achieved the lowest WER results of 15.2% on the validation and 14.3% on the test sets. These results effectively illustrate the Uzbek language dataset for ASR model training.

**Figure 10:**
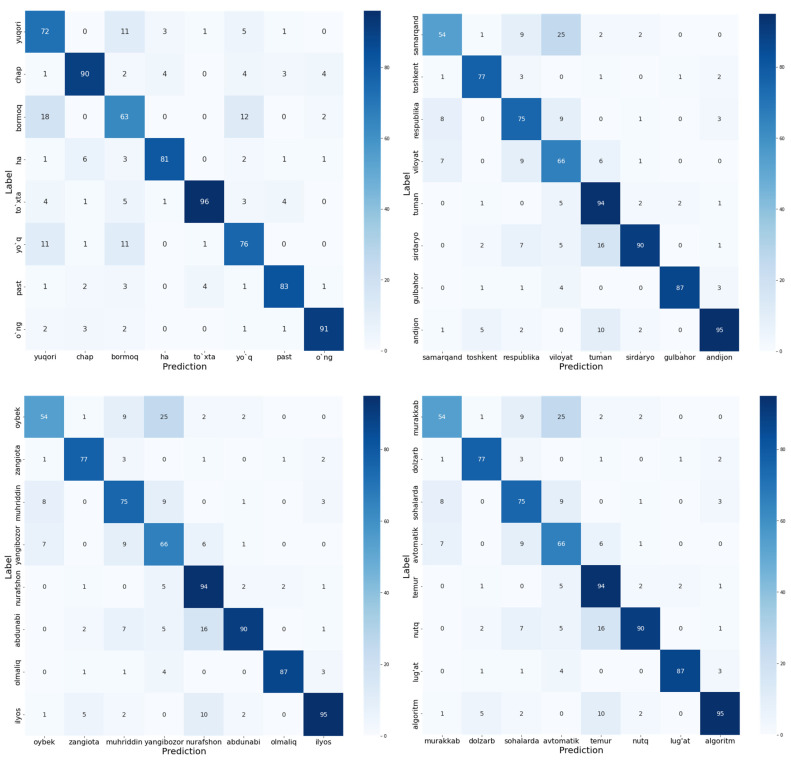
Confusion matrix of actual labels and the prediction of the proposed E2E − T model. The rows represent an actual label and columns represent corresponding prediction results.

Furthermore, we also evaluated the performance of the proposed E2E − T model accuracy using the confusion matrix metric. The confusion matrix compares the actual labels to the model’s predictions. It provides us with a holistic perspective of the proposed model’s performance and the types of errors it makes. The results of confusion matrix evaluation are illustrated in Figure 10. In this evaluation, we used the random 32 words which belong to various types of words such as nouns, verbs, and adjectives.

### 5.5. The Effect of Speech Segment Length

In traditional ASR applications, transcriptionists control the duration of the speech segments. However, in actual ASR applications, automatic segmentation results in substantial variability in the duration over time. Using voice activity detection (VAD), we investigated how segment duration varies between the E2E − T and DNN-HMM systems. Compared to human-produced training data, an automated VAD was beneficial, such as an InaSpeechSegmenter (IS) can produce highly long segments. When the segment duration is changed, the statistical properties of the data are altered, causing a shift in the data distribution. To solve this issue, we came up with a new VAD pipeline called Imp_IS that combines the advantages of IS, which is good at identifying speech, and an energy-based VAD, which controls how long each segment can last. Imp_IS is implemented by applying an energy-based VAD and IS in parallel, resulting in no additional delay. Subsequently, the energy-based VAD segments are aligned with the appropriately long segments from the IS. This study discovered that the suggested Imp_IS VAD produced the best results when the maximum time threshold was set to 15 s. It is worth noting that the automated segmentation was applied to all audio files; hence, Table 9 and Table 10 include the entire Uzbek_Test set with overlapping segments, as opposed to the official Uzbek_Test set listed in Table 5 and Table 6, which only includes non-overlapped segments. Table 9 summarizes the WER (%) of the E2E − T and DNN-HMM findings of the human segmentation (HS), IS segmentation, and suggested Imp_ IS. On the Uzbek_Test and Hidden_Test, the suggested Imp_IS technique increases the IS WER by 25.9% and 17.3%, respectively, and reaches the optimal human segmentation with an absolute difference of only 2.1%. When comparing the results of IS with the segmentation of Imp_IS WER, the influence of the segment duration on DNN-HMM was 2.45% on average. However, when comparing HS with Imp_IS, the DNN-HMM findings were still influenced by the automated segmentation, with absolute differences of 6.7% and 10.2%. Table 10 shows the WER findings of E2E − T and HMM-DNN on the Uzbek_Test set with 0–10 s, 11–20 s, and 21 s and longer to explore the influence of the segmentation duration.

The WER of E2E − T increased by 42.3% compared to the 11–20 s segmentation range, as shown in Table 9. Compared to the 11–20 s range, the WER of DNN-HMM for 21 s or longer segmentations was reduced by only 2%. Compared to the modular ASR system, this demonstrates the significant impact of extremely lengthy segments on the E2E − T system.

## 6. Limitations and Future Work

The main limitation of character-level LM is the difficulty in correctly capturing the context of the word in a sentence. In addition, HMM-DNN models have a modular structure with multiple modules such as acoustic modeling, pronunciation lexicon, and language modeling that are trained individually, resulting in model complexity and the need for more linguistic resources.

In the future, we plan to minimize the current research gaps between humans and machines in the Uzbek ASR system and solve the problem of low resources in various Uzbek language dialects that still have a high error rate. In addition, we plan to extend our approach and conduct a comparative study using low-rank approximation methods.

## 7. Conclusions

In this study, we collected an Uzbek language speech corpus to train the proposed ASR model and presented a comprehensive study by comparing the modular DNN-HMM ASR with the E2E ASR for the Uzbek language. We conducted an error analysis to compare the performance of the most advanced ASR system with that of a linguist and a native speaker. The experiments showed that the machine ASR system performed significantly better than a native speaker. Furthermore, we observed a high similarity between the machine errors and the linguist transcription by applying WER, which has an average performance of 3.5% lower than the language linguist in transcribing the raw Uzbek text. For the Uzbek ASR and its dialects, we also developed an E2E transformer. The proposed E2E transformer achieved the highest results of 14.3% in comparison with the other state-of-the-art models. The experiments have presented in real-life ASR, the variability in the feature period strongly affects the performance of the E2E transformer. A VAD pipeline with a maximum-duration threshold was applied to solve this problem.

## Figures and Tables

**Table 1:** Layer parameters of the proposed network model.

Name of the Layer	Layer Parameters	Number of Layers
Incoming	40 × 1	1
Convolutional	Filter size = 5 × 1, number = 256, step = 1, Dropout = 20%, Activation = Relu	3
Recurrent layer	RNN type = LSTM, layer size = 256, Batch size = 32, optimizer = Adam, Training rate = 0.001, Dropout = 20%, Activation function = Relu	2
Fully contacted layers	Layer size = 256, Dropout = 20%, Activation function = Softmax	1
Transcription layer	CTC Loss function	1

**Table 2:** Contains the specifics of the Common Voice and SpeechOcean datasets. The ‘training’ set trains the ASR model, while the ‘testing’ set tests its accuracy.

Dataset	Total Words	Total Unique Words	Total Duration (h)	Total Utterances	Average Duration (s)	Average Word per Utterances
Common Voice (training) Common Voice (testing) SpeechOcean	497,326	78,604	127.41	117,526	4.27	4
	23,450	14,108	11.69	8840	4.23	5
	406,904	50,256	79.95	63,284	5.93	8
Common Voice (training) + SpeechOcean	904,230	128,860	207.36	180,810	4.80	6

**Table 3:** Specifications of the Uzbek language corpus dataset.

Category of Dataset	Training	Validation	Testing	Total
Duration (h)	180.36	16.2	10.8	207.36
#Utterances	162.02	10.98	7.81	180.81
#Words	811.98k	46.95k	45.3k	904.23k
#Unique Words	90.36k	20.16k	18.34k	128.86k
#Speakers	1082	107	93	1282

**Table 4:** Tuned hyperparameters for the acoustic modeling (AM) and language modeling (LM) transformers based on the grid search.

	Hyperparameters of AM	Hyperparameters of LM
Input	batch-bins: 22,000,000	batch-size: 64
Encoder	12 layers, 8 attention heads/layer	12 layers, 4 attention heads/layer
Decoder	6 layers, 8 attention heads/layer	6 layers, 8 attention heads/layer
dmodel(attention)	256	256
FFN	2048	2048

**Table 5:** The results of the study were obtained using the Uzbek_Test set and the Hidden_Test set, analyzing the WER performance (%) for the E2E transformer (E2E − T) with CTC, Attention, and Attention + CTC, and HMM-DNN.

	DNN-HMM	E2E − T (CTC)	E2E − T (Attention)	E2E − T (CTC+ Attention)
Uzbek_Test	19.4%	20.7%	16.4%	14.3%
Hidden_Test	19.6%	20.8%	17.6%	14.4%

**Table 6:** The E2E − T Transformer and LM rescoring setups were compared (WER % and RT factor). Transcription (TR) and background (BG) texts were used to train the language models.

Methods	Beam 20		Beam 5		Beam 2	
	WER	RT	WER	RT	WER	RT
E2E − T + LSTM − LM (BG + TR)	15.4	4.46	16.9	1.89	16.6	1.87
E2E − T + T − LM (BG + TR)	14.7	6.37	15.7	2.62	15.9	1.96
E2E − T + T − LM (TR)	14.6	6.36	15.4	2.53	15.5	1.71
E2E − T	14.3	4.31	15.2	1.97	15.3	1.48

**Table 7:** The comparison results of the proposed E2E ASR model and other state-of-the-art-results.

Models	Train Accuracy	Test Accuracy	WER
Hidden Markov Model based [61]	85%	69%	31%
Hybrid approach-based model [61]	89%	73%	27%
CNN + BLSTM + CTC loss [61]	91%	75%	25%
Transformer based Model [61]	93%	81%	19%
Our E2E − T (CTC + Attention) model	**96%**	**93%**	**14.3%**

**Table 8:** The CER (%) and WER (%) results of different E2E ASR models were trained using the Uzbek language speech corpus. The impact of language model (LM), speed perturbation (SP), and spectral augmentation (SA) are also reported.

Model	LM	SP	SA	Valid		Test	
				CER	WER	CER	WER
E2E-LSTM	**×**	**×**	×	13.8	43.1	14.0	44.0
	√	×	×	14.9	30.0	14.3	31.4
	√	√	×	13.7	27.6	14.4	30.6
	√	√	√	12.6	24.9	12.0	27.0
DNN-HMM	×	×	×	12.8	34.7	10.2	32.1
	√	×	×	10.3	20.5	8.6	24.9
	√	√	×	6.9	18.8	7.5	23.5
	√	√	√	6.9	19.9	8.1	24.9
RNN-CTC	×	×	×	13.3	35.8	9.7	32.3
	√	×	×	12.2	27.2	9.1	24.3
	√	√	×	10.9	25.1	8.7	23.9
	√	√	√	8.3	24.7	7.9	22.3
E2E − Transformer	×	×	×	12.3	35.2	9.4	31.6
	√	×	×	11.7	25.7	8.7	23.9
	√	√	×	10.7	23.9	8.4	23.0
	√	√	√	9.9	21.4	7.6	21.0
E2E-Conformer	×	×	×	12.7	37.6	10.7	35.1
	√	×	×	11.5	27.5	9.7	26.3
	√	√	×	9.2	21.7	7.5	21.2
	√	√	√	7.8	18.1	5.8	17.4
E2E − T (CTC + Attention)	×	×	×	12.1	33.2	9.8	30.3
	√	×	×	9.6	19.4	7.9	22.7
	√	√	×	6.4	17.9	7.4	20.3
	√	√	√	5.7	15.2	5.41	14.3

**Table 9:** Human segmentation, InaSpeech segmentation, and the proposed improved InaSpeech segmentation are all benchmarked using the E2E − T transformer and the DNN-HMM WER (%) results on two sets: Uzbek Test and Hidden Test.

	Uzbek_Test		Hidden_Test	
	E2E − T	DNN-HMM	E2E − T	DNN-HMM
HS	17.6	24.4	14.4	18.1
IS	41.6	28.7	34.8	25.7
IMP_IS	19.7	31.1	16.5	28.3

**Table 10:** E2E − T transformer and DNN-HMM WER (%) outcomes with segmentation ranges of 0–10 s, 11–20 s, and 21 s or more on the Uzbek_Test set.

	Uzbek_TEST SEGMENTATION		
	0–10 s	11–20 s	21 s or More
E2E − T	18.9	18.4	60.7
DNN-HMM	30.6	27.8	29.9
